# Supplementary material for: Simultaneous Zn2+ tracking in multiple organelles using super-resolution morphology-correlated organelle identification in living cells
Source: Nat Commun. 2021 Jan 4;12:109. doi: 10.1038/s41467-020-20309-7 (PMC7782730; doi:10.1038/s41467-020-20309-7)
Supplement: Supplementary file 1 — Supplementary Information [file 41467_2020_20309_MOESM1_ESM.pdf]

Supplementary Information

Simultaneous Zn^{2+} Tracking in Multiple Organelles using by Super-resolution Morphology-correlated Organelle Identification in Living Cells

Hongbao Fang, Shanshan Geng, Mingang Hao, Qixin Chen, Minglun Liu, Chunyan Liu, Zhiqi Tian, Chengjun Wang, Takanori Takebe, Jun-Lin Guan, Yuncong Chen, Zijian Guo, Weijiang He, Jiajie Diao

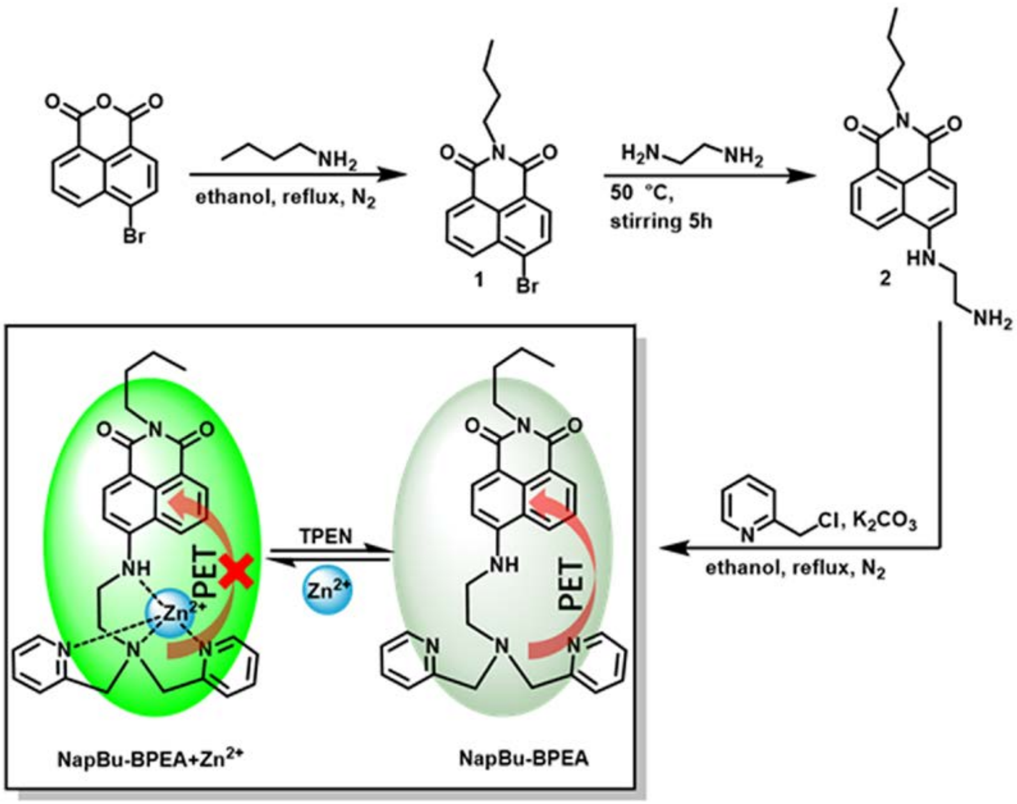

Supplementary Fig. 1 Synthesis of probe NapBu-BPEA and its fluorescent Zn^{2+} sensing behavior.

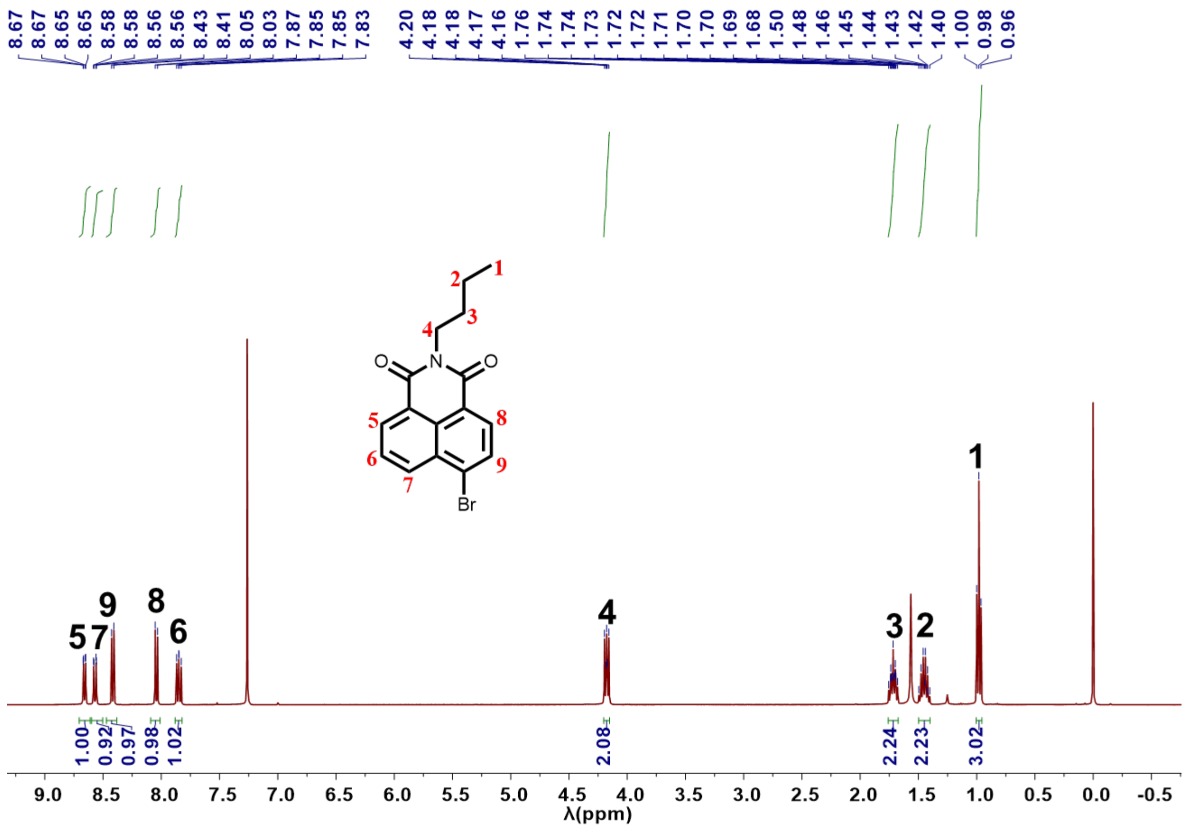

Supplementary Fig. 2 ^{1}H NMR spectrum of compound 1 in $CDCl_3$ (400 MHz).

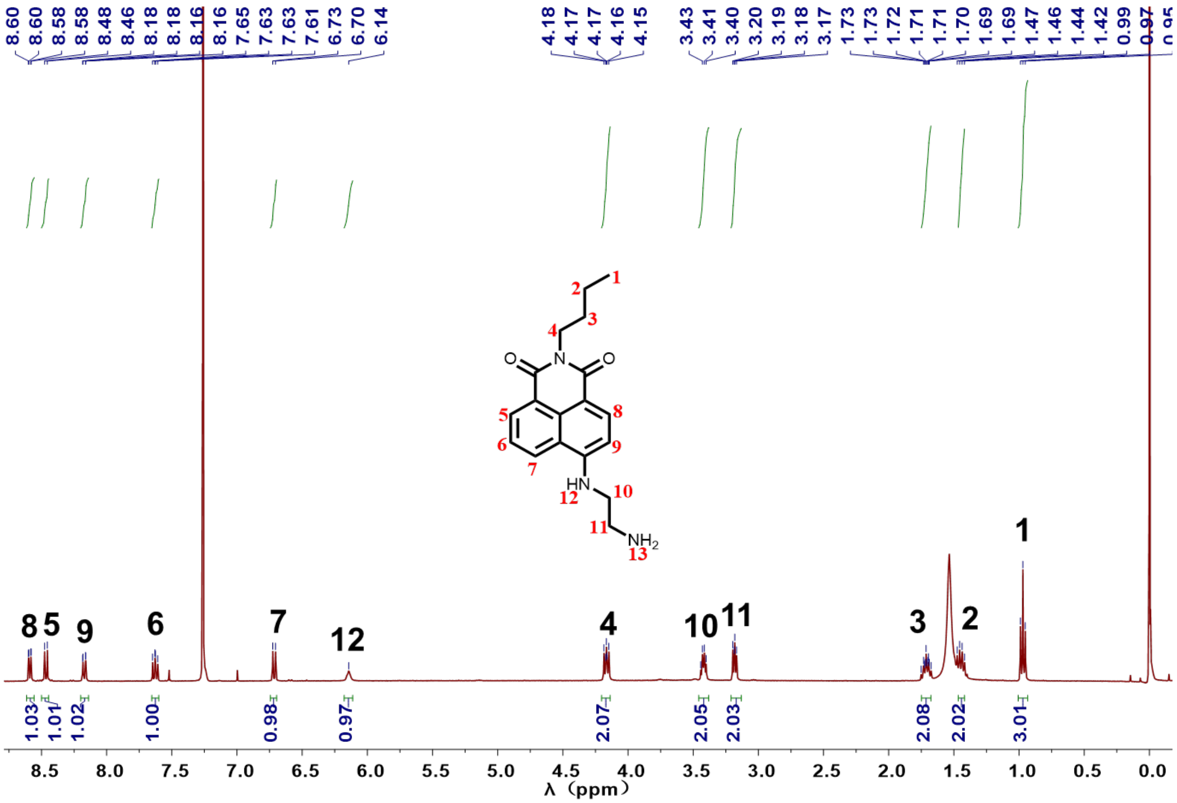

Supplementary Fig. 3 ^{1}H NMR spectrum of compound 2 in $CDCl_3$ (400 MHz).

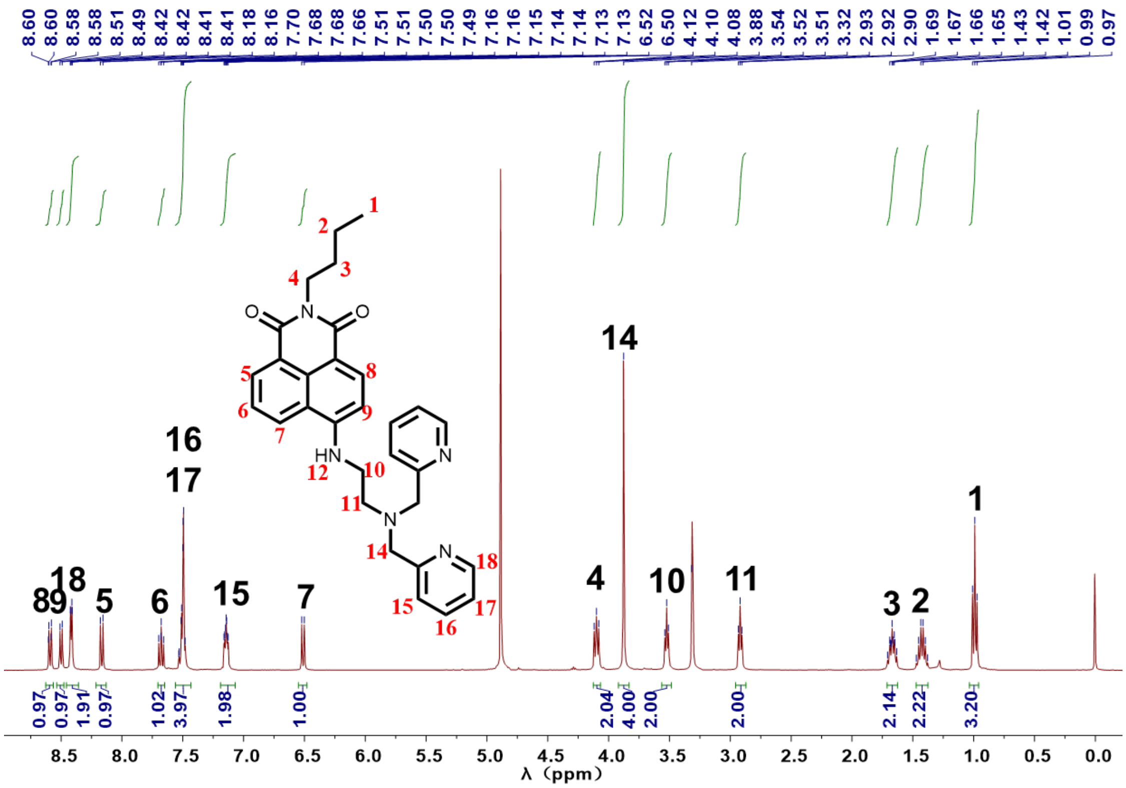

Supplementary Fig. 4 ^{1}H NMR spectrum of NapBu-BPEA in methanol-d_4 (400 MHz).

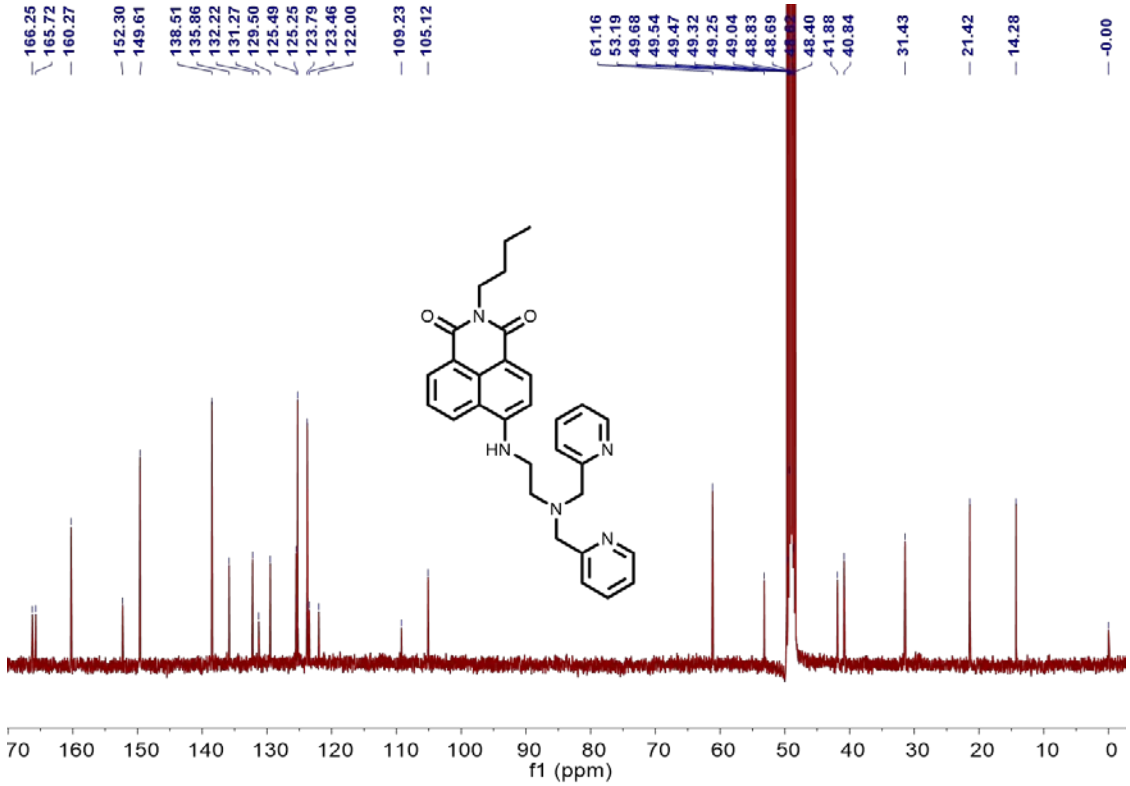

Supplementary Fig. 5 ^{13}C NMR spectrum of NapBu-BPEA in methanol-d_4 (101 MHz).

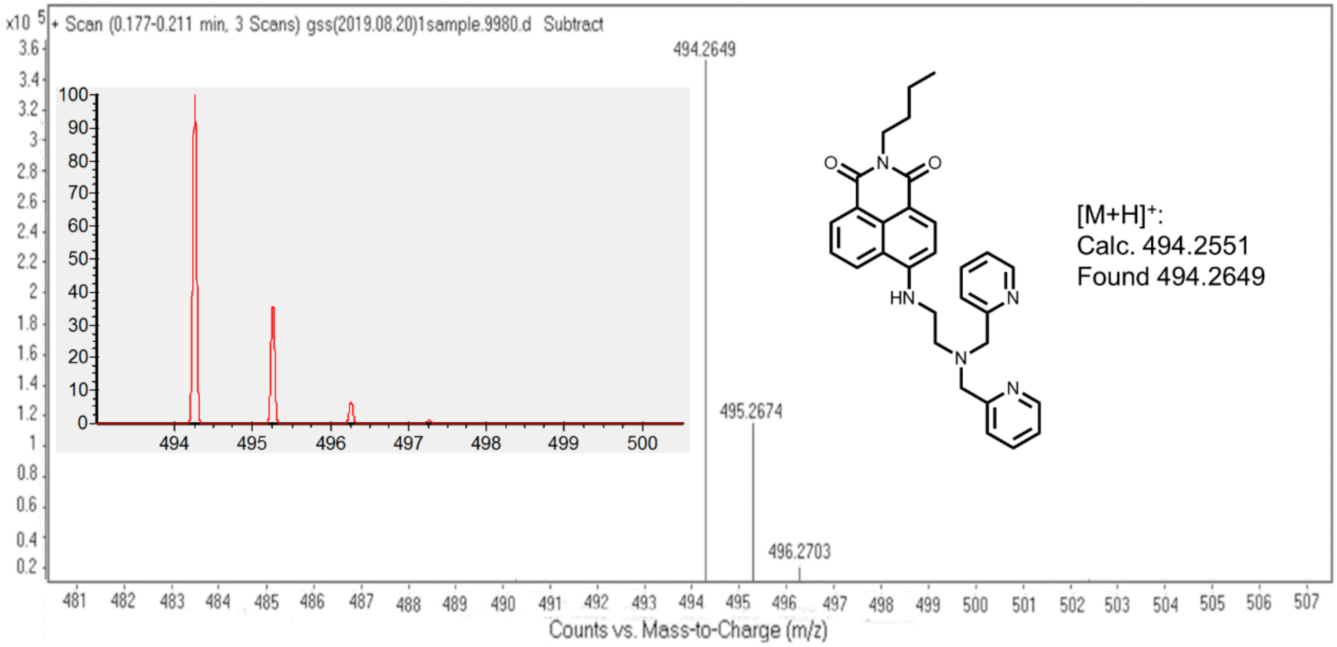

Supplementary Fig. 6 HR-MS spectrum of NapBu-BPEA. Inset is the simulated isotopic distribution pattern of [NapBu-BPEA+H]$^+$.

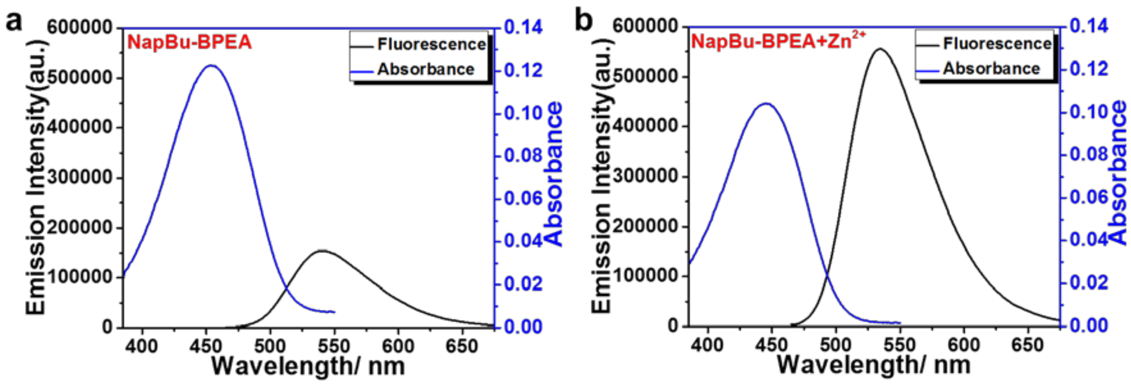

Supplementary Fig. 7 Fluorescence (black line) and absorbance (blue line) spectra of NapBu-BPEA in absence (a) and presence (b) of Zn^{2+} (1 eq) in HEPES buffer (50 mM, 100 mM KNO_3, 1: 9, pH 7.2) containing 10% DMSO (v/v).

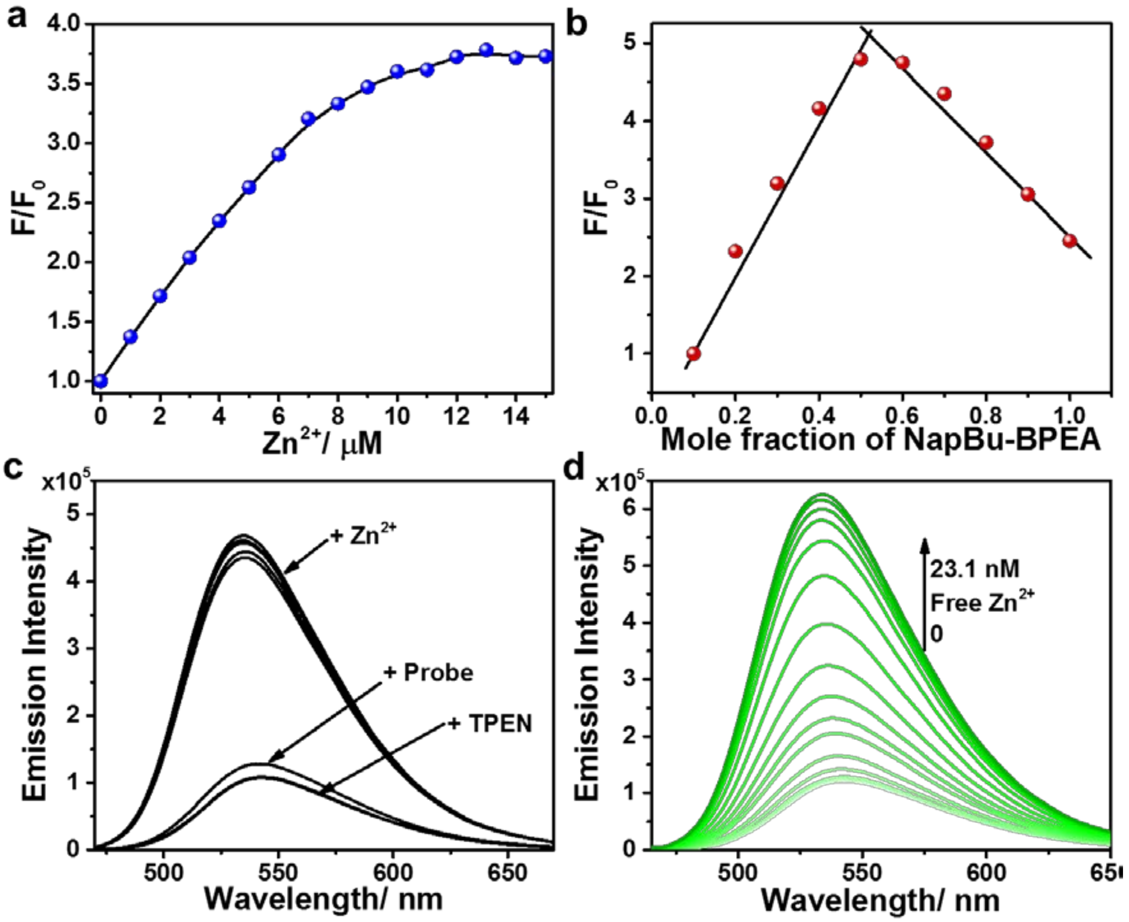

Supplementary Fig. 8 (a) Zn^{2+} titration profile of NapBu–BPEA (10 μM) based on emission at 540 nm in HEPES buffer (50 mM; 100 mM KNO_3; pH 7.2) containing 10% DMSO (v/v); (b) Job's plot of NapBu–BPEA according to emission at 540 nm; (c) fluorescence spectra of NapBu-BPEA (10 μM) determined in the cycles of Zn^{2+} addition (1 eq) and subsequent TPEN (1 eq) treatment; (d) fluorescence emission spectra of NapBu-BPEA (10 μM) determined in a series of HEPES buffer (50 mM, 100 mM KNO_3, 10% DMSO, 10 mM EGTA, pH=7.21) containing $[Zn^{2+}]_{total}$ from 0 to 9 mM. All determination was performed upon excitation at 450 nm.

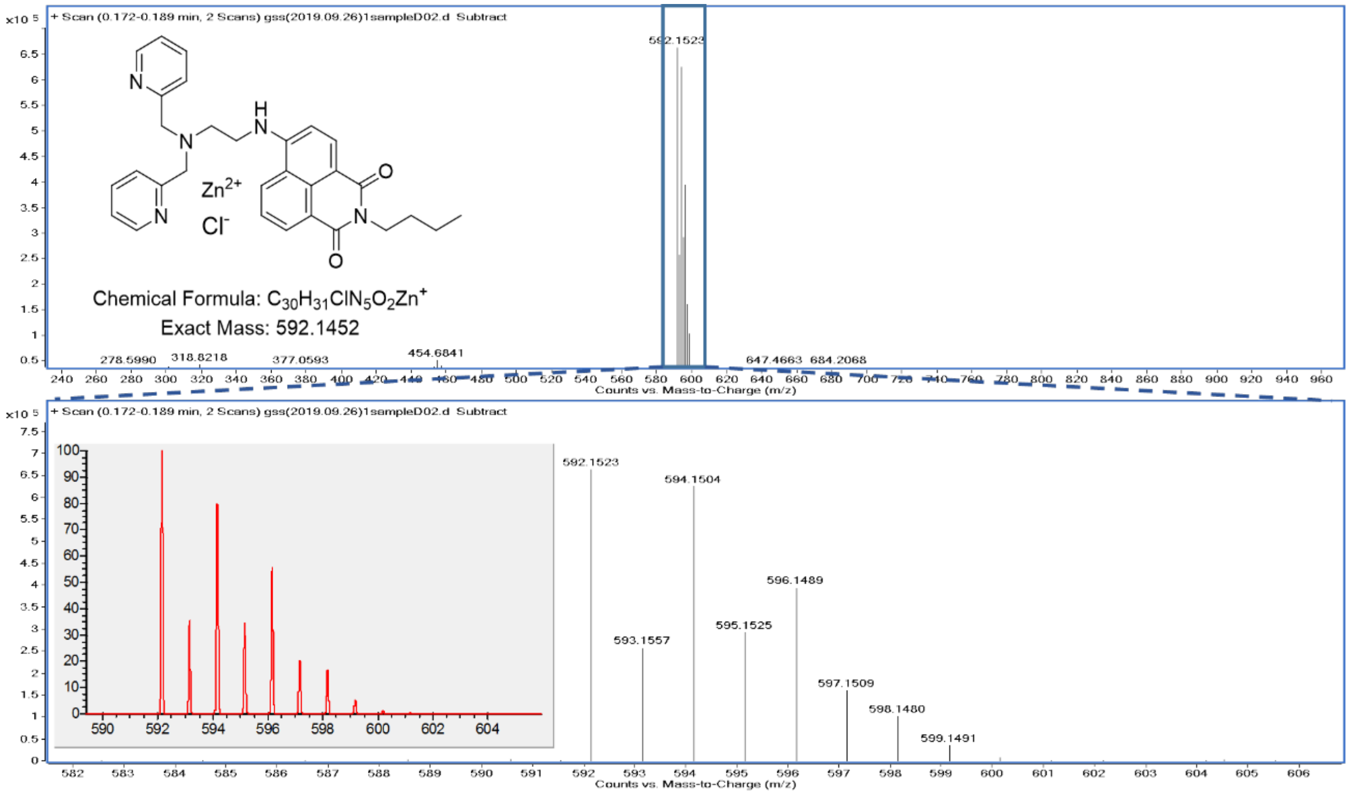

Supplementary Fig. 9 HR-MS spectra (positive mode) of NapBu-BPEA determined after adding 1 eq Zn^{2+}. Inset is the simulated isotopic distribution pattern of $[NapBu–BPEA+Zn+Cl]^{+}$.

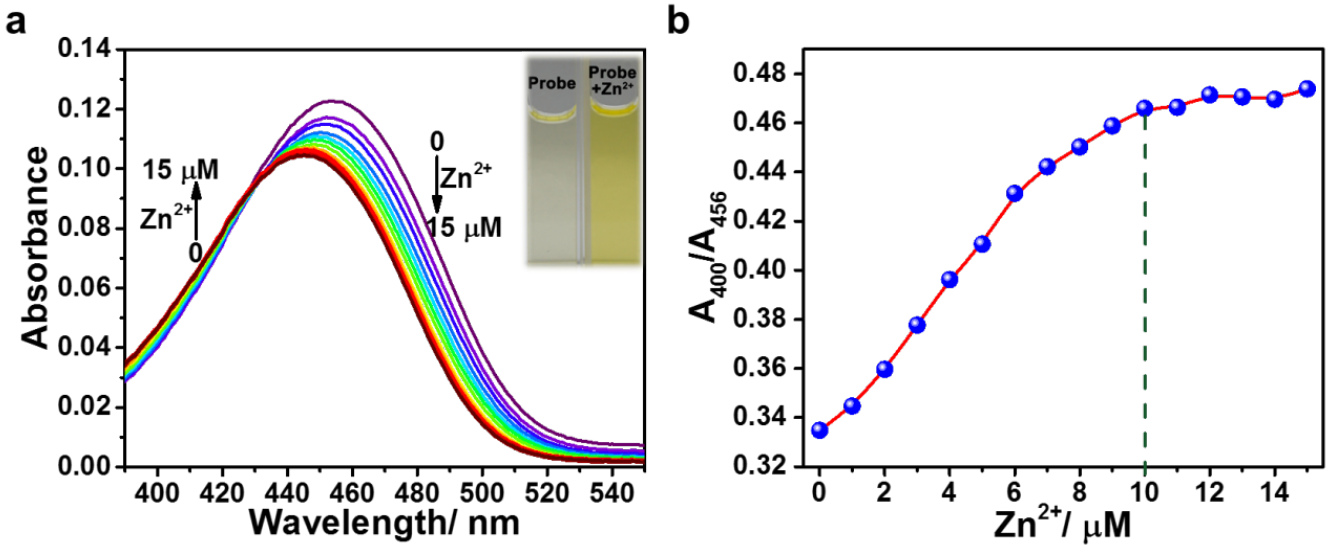

Supplementary Fig. 10 (a) Absorption spectra of NapBu-BPEA (10 μM) determined upon Zn^{2+} titration HEPES buffer (50 mM; 100 mM KNO_3; pH 7.2) containing 10% DMSO (v/v). Inset: photograph of NapBu-BPEA solution before and after Zn^{2+} addition (1 eq.) in ambient condition; and the related titration profile based on the ratio of absorbance at 400 nm to that at 458 nm.

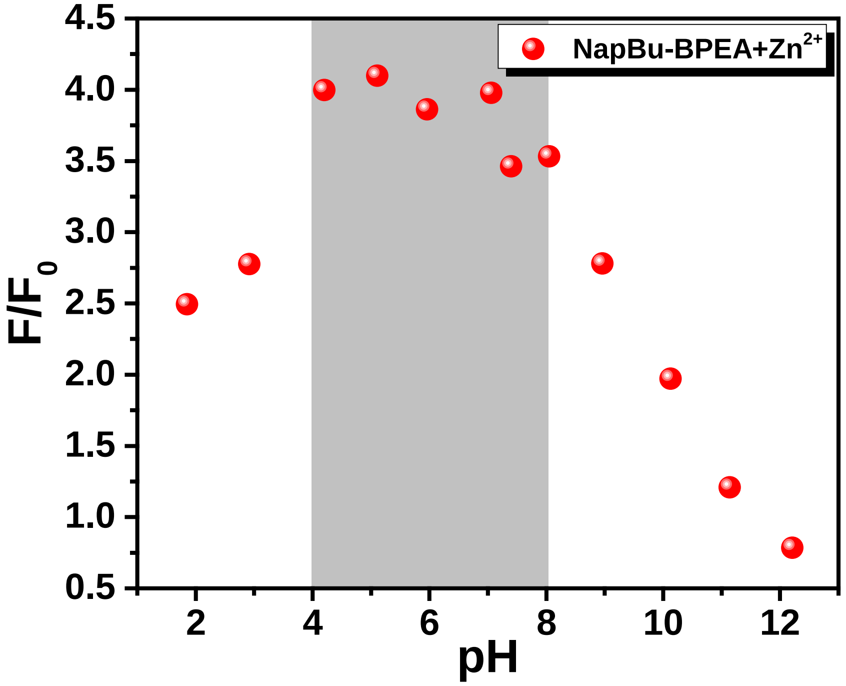

Supplementary Fig. 11 pH-titration plot of NapBu-BPEA (10 μM) in the present of 1 eq Zn^{2+} based on the emission at 540 nm. λ_{ex}= 450 nm, λ_{em}= 540 nm.

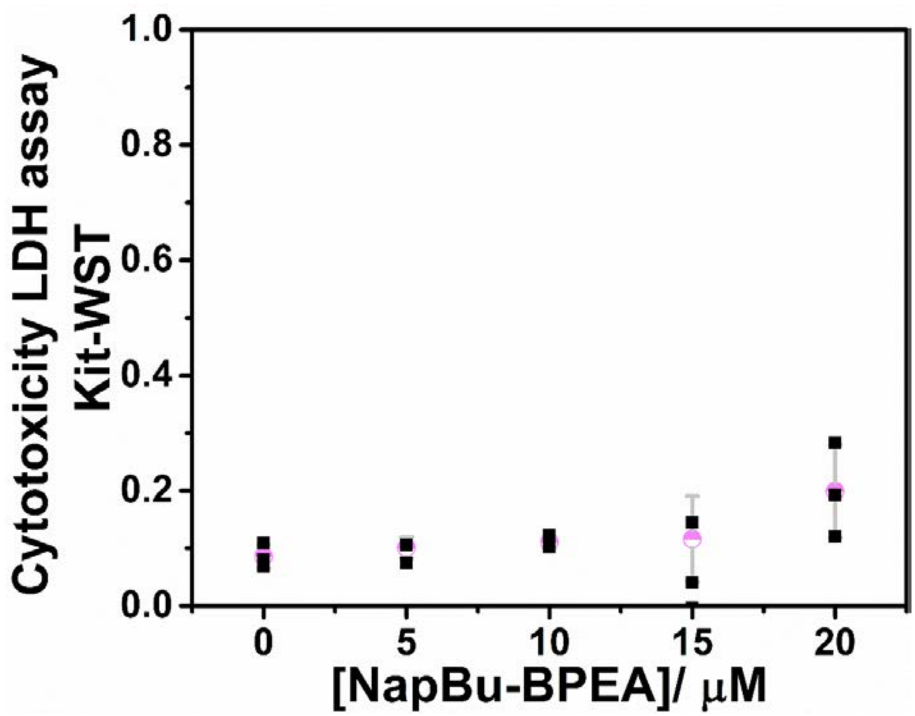

Supplementary Fig. 12 Cytotoxicity of NapBu-BPEA against HeLa cells determined as the activity of lactate dehydrogenase released into the medium using a water-soluble tetrazolium (WST) kit at different probe concentration (24 h). $n = 3$ biologically independent experiments per group, mean ± SD.

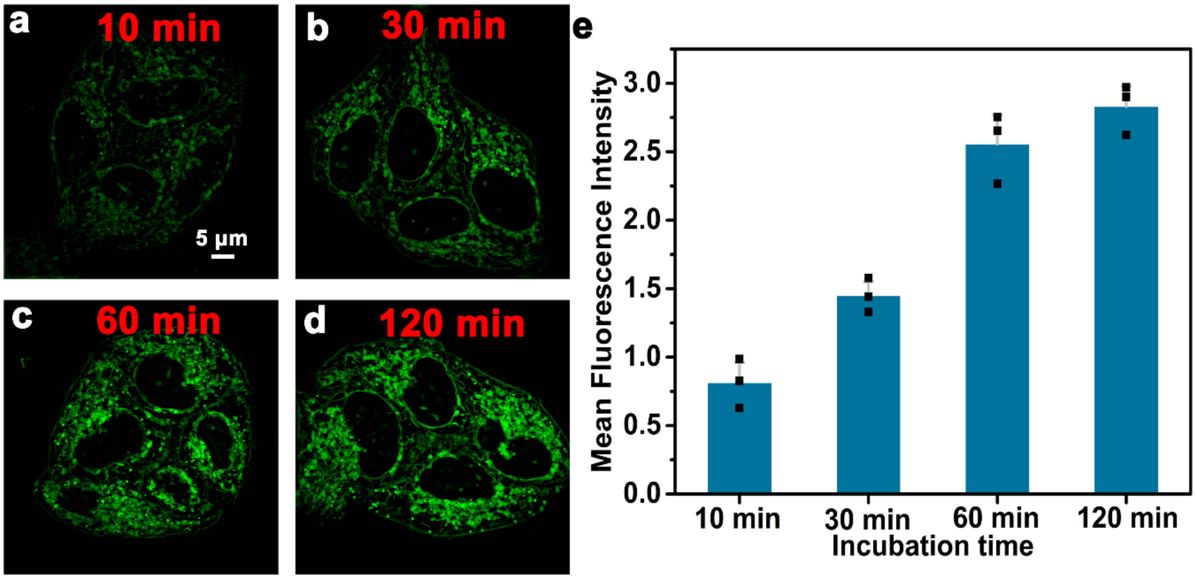

Supplementary Fig. 13 SIM images of HeLa cells recorded at different NapBu-BPEA (10 μM) incubation time. n = 3 biologically independent experiments, mean ± SD. Scale bar, 5 μm.

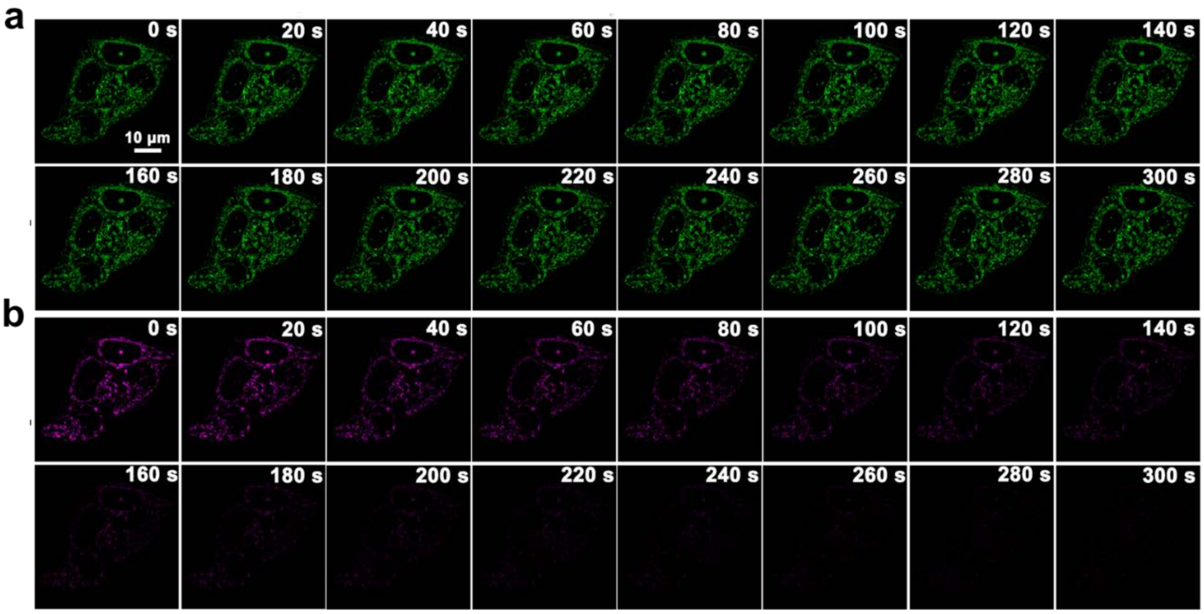

Supplementary Fig. 14 SIM images of HeLa cells costained by NapBu-BPEA (10 μM, 1 h, 37°C) and MitoTracker Deep Red (1 μM, 30 min, 37°C) recorded in (a) green channel for NapBu–BPEA and (b) magenta channel for MTDR at different irradiation time. Scale bar, 10 μm.

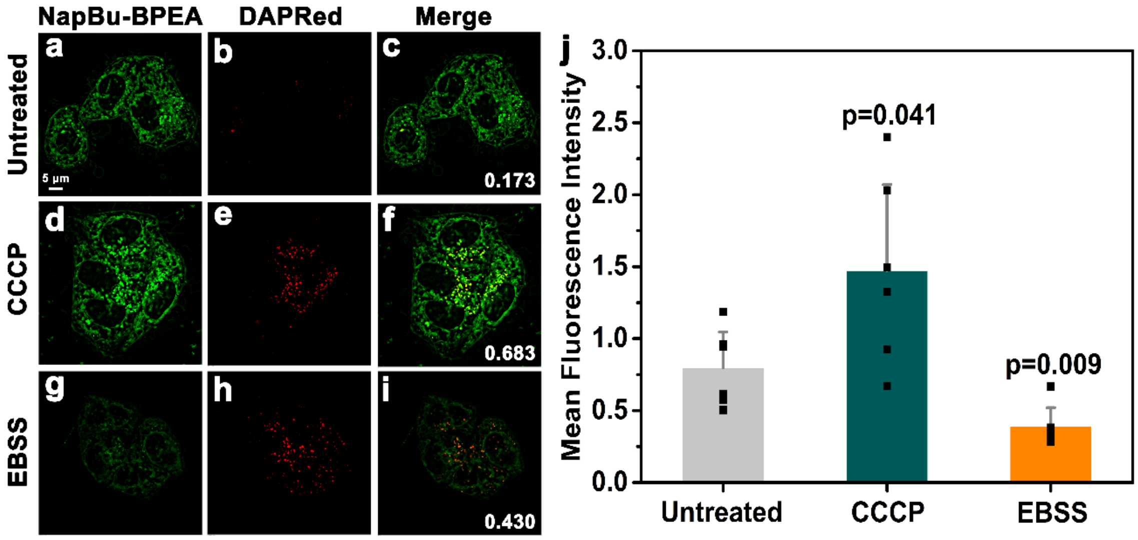

Supplementary Fig. 15 SIM images of the DAPRed (1 μM, 30 min, 37°C) stained HeLa cells further treated with CCCP (10 μM, 24 h, 37°C) or EBSS (24 h, 37°C). NapBu–BPEA (10 μM, 1 h, 37°C) staining was performed prior to imaging. (a, d, g) Green channel from NapBu–BPEA-incubated HeLa cells; (b, e, h) Red channel from DAPRed-incubated HeLa cells; (c, f, i) merged images of red and green channel images; (j) histogram the recorded average fluorescence intensity from (a, d, g). *n* = 6 biologically independent experiments per group, mean ± SD, the statistical differences between the experimental groups were analyzed by one way ANOVA test. When $p < 0.05$, it was considered to have statistical significance. Scale bar, 5 μm.

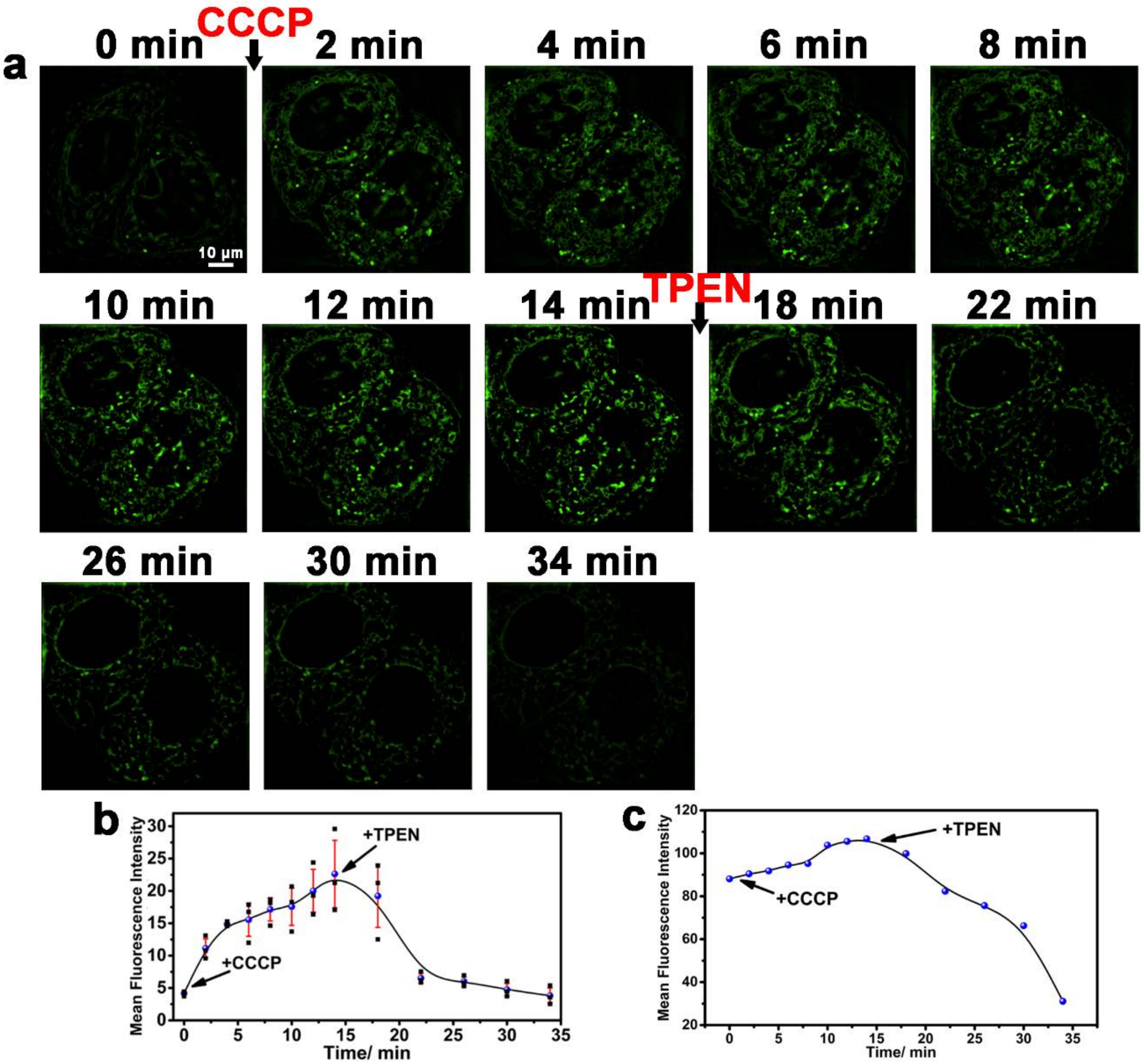

Supplementary Fig. 16 (a) Time-lapse SIM images of the NapBu-BPEA-stained HeLa cells recorded during incubation with 20 μM CCCP. TPEN (100 μM) treatment was administrated after 14 mins of CCCP treatment, and the corresponding temporal profiles of mean fluorescence intensity inside cells (b) and inside dot-shaped organelles (c). (b) n = 3 biologically independent experiments per group, mean ± SD. Scale bar, 10 μm.

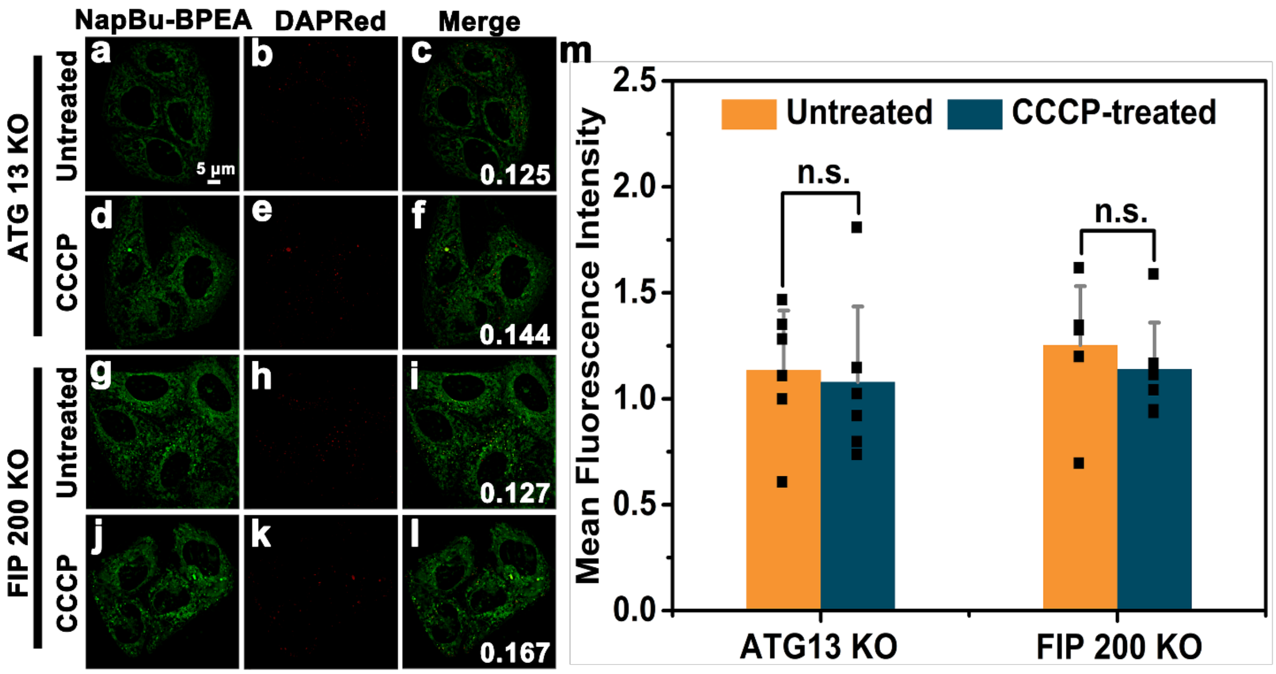

Supplementary Fig. 17 SIM images of ATG13 KO (a-f) and FIP 200 KO (g-l) HeLa cells stained by NapBu–BPEA and DAPRed with or without the CCCP (10 μM, 24 h, 37°C) treatment. (a, d, g, j) cell images from the green channel for NapBu–BPEA (10 μM, 1 h, 37°C) fluorescence; (b, e, h, k) cell images from the red channel for DAPRed (1 μM, 30 min, 37°C) fluorescence; (c, f, I, l) merged images of red and green channel images; (m) average fluorescence intensity recorded in the images from green channel. *n* = 6 biologically independent experiments per group, mean ± SD, the statistical differences between the experimental groups were analyzed by double-tailed student's t-test, $p > 0.05$ shows no significant difference (n.s.). Scale bar, 5 μm.

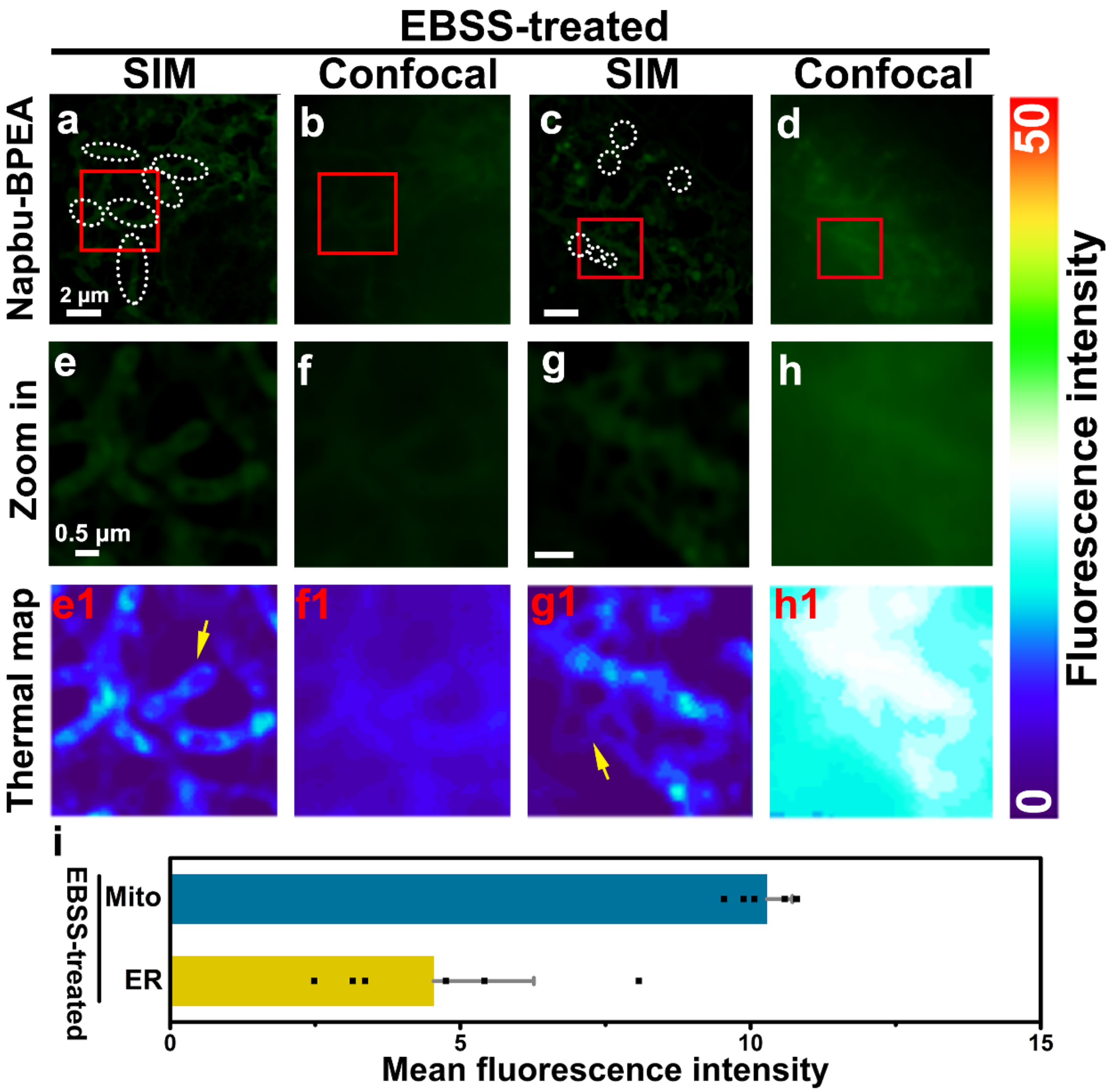

Supplementary Fig. 18 Subcellular Zn^{2+} level changes under EBSS-treatment in super-resolution imaging and confocal imaging. (a, c) The SIM images of NapBu-BPEA-loaded HeLa cells with EBSS treatment; (b, d) The confocal images of NapBu-BPEA-loaded Hela cells with EBSS treatment; (e–h) zoom in images on frames from (a–d); (e1–h1) fluorescence intensity thermal images constructed respectively from (e–h); (i) mean fluorescence intensity for ROIs in (mitochondria: a; endoplasmic reticula: c). *n* = 6 biologically independent experiments per group, mean ± SD. Scale bar: 2 μm, zoom in images scale bar: 0.5 μm.

Supplementary Table 1 Photophysical properties of free NapBu-BPEA and its Zn^{2+} complex formed with 1 eq Zn^{2+} in HEPES buffer (50 mM; 100 mM KNO_3; 1: 9, v/v; pH 7.2).

	λ_{abs}/ nm	ε_{max}/ 10^4 $M^{-1}cm^{-1}$	λ_{em}/ nm	Φ_f
NapBu-BPEA	545	1.226	540	0.32
NapBu-BPEA+Zn^{2+}	445	1.044	535	0.80

Supplementary Table 2 Selected parameters for the calculated singlet state energy level and triplet state energy level of NapBu-BPEA and Naph-BPEA. [a]

**Compound**	**States**	**Electronic transition**	**Energy, eV/λ, nm**	***f*[b]**	**Composition[c]**	**CI[d]**
NapBu-BPEA	Singlet State	S0→S1	3.26/380	0.2329	H→L	0.6936
		S0→S2	3.54/350	0.0002	H-1→L	0.6887
		S0→S3	3.65/340	0.0004	H-2→L	0.7048
	Triplet State	S0→T1	2.22/557	0.0000	H→L	0.6754
		S0→T2	3.38/366	0.0000	H-2→L	0.6592
		S0→T3	3.41/364	0.0000	H-3→L	0.4141
Naph-BPEA	Singlet State	S0→S1	3.18/390	0.2523	H→L	0.6941
		S0→S2	3.64/340	0.0001	H-3→L	0.6332
		S0→S3	3.76/329	0.0001	H-1→L	0.7051
	Triplet State	S0→T1	2.14/579	0.0000	H→L	0.6846
		S0→T2	3.37/368	0.0000	H-5→L	0.4138
		S0→T3	3.50/354	0.0000	H-3→L	0.6183

[a] Parameters were calculated by TD-DFT//B3LYP/6-31G(d), based on the optimized ground-state geometries using the method DFT//B3LYP/6-31G(d).

[b] Oscillator strength.

[c] H, HOMO (highest occupied molecular orbital) and L, LUMO (lowest unoccupied molecular orbital).

[d] Coefficient of the wavefunction for each excitations.

Supplementary Table 3 The K_d of organelle-targeted Zn^{2+} probes.

**Probes**	**K_d/ nM**	**Organelles**	**Cells**	**References**
RhodZin-3 AM	~65	Mitochondria	Neurons	S. L. Sensi et al., *P. Natl. Acad. Sci. USA* **2003**, *100*, 6157.
ZP1-TPP	0.6	Mitochondria	HeLa cells	W. Chyan et al., *P. Natl. Acad. Sci. USA* **2014**, *111*, 143.
DQZn4	0.2	Lysosome	NIH 3T3 cells	L. Xue, et al., *Inorg. Chem.* **2012**, *51*, 10842.
LysoDPP-C4	1.91	Lysosome	HeLa cells	C. Du et al., *Chem. Sci.* **2019**, *10*, 5699.
Probe 9	3.5	ER	HeLa cells	L. Fang et al., *Chem. Sci.* **2019**, *10*, 10881.
SZnC	1.7	Golgi	HeLa cells	H. Singh et al., *Chem. Commun.* **2015**, *51*, 12099.
